# Controlled Release of Hydrogen‐Carrying Perfluorocarbons for Ischemia Myocardium‐Targeting ^19^F MRI‐Guided Reperfusion Injury Therapy

**DOI:** 10.1002/advs.202304178

**Published:** 2023-08-18

**Authors:** Chaoqun Nie, Rong A, Jing Wang, Shuang Pan, Rentong Zou, Bin Wang, Shuiqing Xi, Xiaojian Hong, Meifang Zhou, Haoyu Wang, Mengshu Yu, Lina Wu, Xilin Sun, Wei Yang

**Affiliations:** ^1^ Department of Cardiology The Fourth Hospital of Harbin Medical University 150000 Harbin P. R. China; ^2^ Department of Nuclear Medicine The Fourth Hospital of Harbin Medical University 150000 Harbin P. R. China; ^3^ NHC Key Laboratory of Molecular Probe and Targeted Theranostics Molecular Imaging Research Center (MIRC) of Harbin Medical University 150000 Harbin P. R. China

**Keywords:** hydrogen gas, myocardial ischemia‐reperfusion injury, NLRP3 inflammasome, perfluorocarbon, precision medicine

## Abstract

Hydrogen gas is recently proven to have anti‐oxidative and anti‐inflammation effects on ischemia‐reperfusion injury. However, the efficacy of hydrogen therapy is limited by the efficiency of hydrogen storage, targeted delivery, and controlled release. In this study, H_2_‐PFOB nanoemulsions (NEs) is developed with high hydrogen loading capacity for targeted ischemic myocardium precision therapy. The hydrogen‐carrying capacity of H_2_‐PFOB NEs is determined by gas chromatography and microelectrode methods. Positive uptake of H_2_‐PFOB NEs in ischemia‐reperfusion myocardium and the influence of hydrogen on ^19^F‐MR signal are quantitatively visualized using a 9.4T MR imaging system. The biological therapeutic effects of H_2_‐PFOB NEs are examined on a myocardial ischemia‐reperfusion injury mouse model. The results illustrated that the developed H_2_‐PFOB NEs can efficaciously achieve specific infiltration into ischemic myocardium and exhibit excellent antioxidant and anti‐inflammatory properties on myocardial ischemia‐reperfusion injury, which can be dynamically visualized by ^19^F‐MR imaging system. Moreover, hydrogen burst release induced by low‐intensity focused ultrasound (LIFU) irradiation further promotes the therapeutic effect of H_2_‐PFOB NEs with a favorable biosafety profile. In this study, the potential therapeutic effects of H_2_‐PFOB NEs is fully unfolded, which may hold great potential for future hydrogen‐based precision therapeutic applications tailored to ischemia‐reperfusion injury.

## Graphical abstract

Schematic illustration of hydrogen therapy strategy and mechanisms of enhanced therapeutic effect of H_2_‐PFOB NEs combined with LIFU. The synthesized H_2_‐PFOB NEs with high biosafety and excellent hydrogen carrying capacity, exhibited stronger anti‐oxidant and anti‐pyroptosis effects and exerted excellent therapeutic effect on I/R injury. In addition, H_2_‐PFOB NEs can passively infiltrate into ischemic myocardial tissue, exert further enhanced therapeutic effects when combined with LIFU, realizing the effective release and utilization of hydrogen, thus facilitating the purpose of precise treatment of I/R injury.

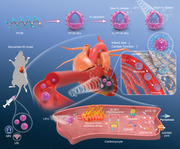

## 1 Introduction

Cardiovascular disease (CVD) is the leading cause of mortality and morbidity globally, and ischemic heart disease (IHD) accounts for approximately half of CVD deaths.^[^ ^1^ ^]^ Revascularization therapy is the most commonly used treatment method for IHD, however, ischemia‐reperfusion (I/R) injury caused by revascularization therapy significantly affects the prognosis of IHD.^[^ ^2^ ^]^ The underlying mechanisms of I/R injury are complex and primarily involve calcium overload, mitochondrial oxidative stress damage, and the release of a large number of inflammatory factors.^[^ ^3^ ^]^ Thus, new therapeutic strategies are needed to mitigate above influential factors in myocardial I/R injury tissue to improve the clinical outcomes of IHD patients.

Hydrogen therapy is a promising emerging treatment based on molecular hydrogen (H_2_) as a new type of safe and effective therapeutic agent.^[^ ^4^ ^]^ In 2007, Ohta et al. found that hydrogen can selectively reduce toxic hydroxyl free radicals to alleviate cerebral I/R injury.^[^ ^5^ ^]^ In the past decade, hydrogen molecules have been found to have highly effective therapeutic effects on a series of oxidative stress and inflammatory diseases, such as arteriosclerosis, cancer, arthritis and diabetes.^[^ ^6^ ^]^ These studies add to a growing body of evidence showing that hydrogen has important clinical implications and high translational potential.^[^ ^7^ ^]^ However, the efficacy of hydrogen‐based treatment of myocardial infarction is limited by the efficiency of its targeted delivery and controlled release. Direct inhalation of hydrogen gas is convenient but generally accompanied by multiple drawbacks, such as low H_2_ concentration in the target area and the potential risk of explosion. In addition, the therapeutic efficacy of hydrogen in water or saline solution is limited by the low solubility and high diffusivity of hydrogen gas. The emergence of nanomaterial‐based platforms provides a promising alternative strategy for hydrogen delivery, and a variety of nanomaterials with hydrogen storage and hydrogen‐producing ability have already been developed for the treatment of various diseases.^[^ ^8^ ^]^ However, current hydrogen‐carrying nanomaterials present important limitations for clinical transformation, particularly the biosafety profiles of some inorganic or hybrid materials, which challenges their further development for clinical applications.^[^ ^9^ ^]^ Thus, the development of hydrogen carriers with outstanding clinical translational potential would be a significant step forward in the treatment of myocardial I/R injury.

Perfluorinated compounds (PFCs) are inert organic compounds that improve tissue oxygenation due to their high affinity for oxygen and perfect biocompatibility.^[^ ^10^ ^]^ These compounds are widely used for several clinical applications, such as artificial blood substitution, organ preservation, ultrasound imaging, and ^19^F magnetic resonance imaging.^[^ ^11^ ^]^ In addition, perfluorooctylbromide nanoemulsions (PFOB NEs) have large solubilization capacity for oxygen via van der Waals interactions,^[^ ^12^ ^]^ and the release of gas contents in PFOB NEs could be facilitated by low‐intensity focused ultrasound (LIFU) at targeted site with excellent biosafety profile due to its low intensity and low frequency ultrasonic characteristics.^[^ ^13^ ^]^ However, it remains unclear whether PFOB NEs may also be used to dissolve hydrogen gas and exert biological effects.

**Figure 1:**
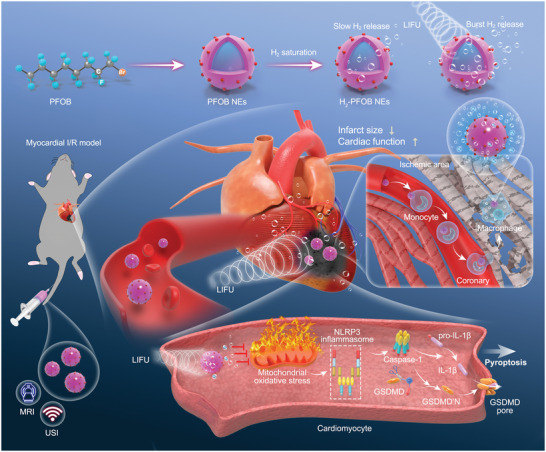
Schematic illustration of hydrogen therapy strategy and mechanisms of enhanced therapeutic effect of H_2_‐PFOB NEs combined with LIFU. We synthesized H_2_‐PFOB NEs with high biosafety, and the excellent hydrogen carrying capacity of PFOB NEs was confirmed by gas chromatograph and microelectrode for hydrogen concentration determination. We for the first time revealed its excellent therapeutic effect on I/R injury and found that H_2_‐PFOB NEs has stronger anti‐oxidant and anti‐pyroptosis effects than H_2_‐water. Remarkably, H_2_‐PFOB NEs can passively infiltrate into ischemic myocardial tissue, this may be an important reason to enhance the therapeutic effect in addition to the high hydrogen loading capacity of H_2_‐PFOB NEs, and the combination with LIFU further enhances its therapeutic effect, realizing the effective release and utilization of hydrogen, thus facilitating the purpose of precise treatment of I/R injury. PFOB NEs, Perfluorooctylbromide nanoemulsions; LIFU, low‐intensity focused ultrasound; NLRP3, Nucleotide‐binding oligomerization domain 3; GSDMD, Gasdermin D.

In this study, we report a novel therapeutic approach with high clinical translational potential for the treatment of myocardial I/R injury therapy based on the multifunctional advantages of a perfluorocarbon nanoplatform (**Figure** 1).

## 2 Results

### 2.1 Synthesis and Characterization of H2‐PFOB NEs

**Figure 2:**
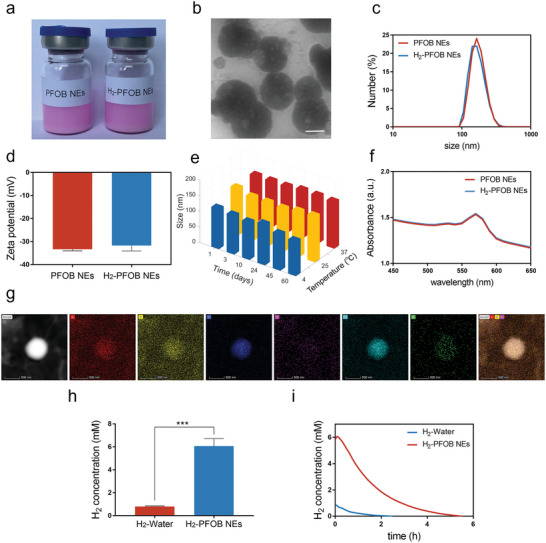
Synthesis and characterization of H_2_‐PFOB NEs. a) Representative image of the H_2_‐PFOB NEs. b) TEM image of H_2_‐PFOB NEs. Scale bar 100 nm. c) DLS data of PFOB NEs and H_2_‐PFOB NEs NEs. d) Zeta potential of PFOB NEs and H_2_‐PFOB NEs. e) DLS of H_2_‐PFOB NEs at different temperatures and time (Days). f) UV‐vis spectra of PFOB NEs and H_2_‐PFOB NEs. g) H_2_‐FPOB TEM mapping. h) Comparison of H_2_ concentration of H_2_‐Water and H_2_‐PFOB NEs. i) Residual H_2_ concentration of H_2_‐Water and H_2_‐PFOB NEs with increasing time. Data are shown by mean ± SD.

PFOB NEs were synthesized and then hydrogenated by hydrogen absorption (**Figure** 2a). H_2_‐PFOB NEs are spherical, small sized (DLS ≈150 nm) and uniformly dispersed, as revealed by TEM (Figure 2b,c). The zeta potential of H_2_‐PFOB NEs (ζ potential ≈−30 mV) is consistent with that of un‐hydrogenated PFOB NEs (Figure 2d). Moreover, DLS measurements showed that H_2_‐PFOB NEs maintain a similar size at different temperatures even after long periods of time, revealing good reproducibility and stability (Figure 2e). The Rhodamine B absorbance peak of H_2_‐PFOB NEs and PFOB NEs measured by UV‐vis is ≈575 nm (Figure 2f). Elemental analysis showed that C, N, F, O, P, and Br are evenly distributed in PFOB NEs (Figure 2g). To determine the hydrogen‐carrying capacity of PFOB NEs, chromatography and hydrogen microelectrodes were used to measure hydrogen concentration. As shown in Figure 2h, PFOB NEs have an excellent hydrogen‐carrying capacity of ≈ 6 mM, which is about eight times higher than that of water. Moreover, the release of hydrogen in H_2_‐PFOB NEs is slower when compared with H_2_‐water, indicating that H_2_‐PFOB NEs can produce hydrogen for long periods and are more conducive to hydrogen storage and utilization (Figure 2i).

### 2.2 Behavior and Mechanism of H_2_‐PFOB NEs Targeted Delivery to Ischemic Myocardium

**Figure 3:**
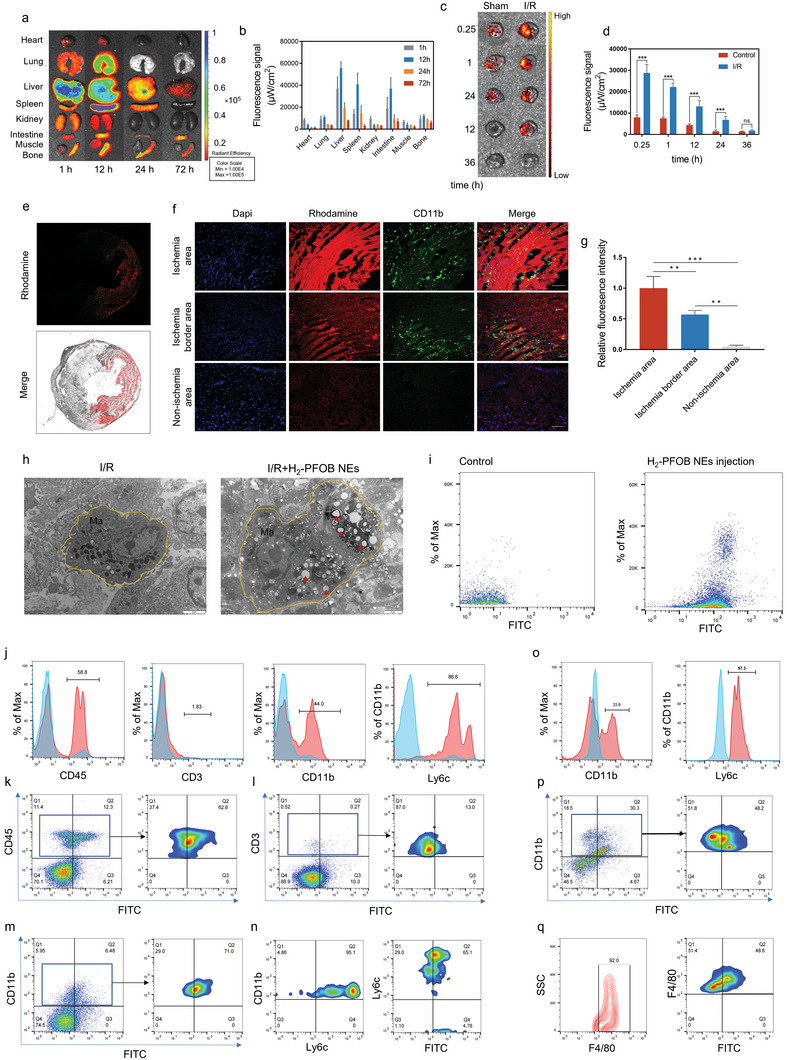
Macrophage mediated targeting of ischemic myocardium of H_2_‐PFOB NEs. a) Representative IVIS images of different times. b) Quantification of IVIS fluorescence intensity, n = 5. c) Representative IVIS images of Sham and I/R mice heart at different time points after injection. d) Quantification of IVIS fluorescence intensity, n = 5. e–g). Immunofluorescence staining of myocardial tissue of I/R mice after injection of H_2_‐PFOB NEs (×20, scale bar 50 µm). h) TEM images of myocardial microtissues (×10 k, scale bar 100 nm). Yellow circles represent macrophages and the red triangle points to the lysosome. i) Peripheral blood mononuclear cells (PBMCs) from a control mouse and a mouse subjected to myocardial infarction and treated with FITC‐labeled H_2_‐PFOB NEs were analyzed for FITC fluorescence by flow cytometry. j) Histograms display specifically stained leukocytes from control (blue) and I/R(red) mouse, leukocytes from I/R mouse was gated on FITC+ cells. Numbers indicate the percentage of FITC+ cells expressing the specific cell marker. Max, maximum. k–m) Gated on WBLs and specific cell markers, which shows FITC+ fluorescence from I/R mouse injected with FITC‐labeled H_2_‐PFOB NEs. n) The percentage of Ly6C positive cells in CD11b^+^ cell population, and the percentage of Ly6C^+^ FITC^+^ in CD11b+Ly6C^+^ cell population. o) Flow cytometry analysis of specifically stained single cell suspension from control (blue) and I/R(red) mouse heart, cells form I/R mouse was gated on FITC^+^ cells. p) Gated on specific cell markers, which shows CD11b+FITC+ fluorescence from I/R mouse injected with FITC‐labeled H_2_‐PFOB NEs. q) The percentage of F4/80 positive cells in CD11b^+^ cell population, and the percentage of F4/80^+^ FITC^+^ in CD11b^+^F4/80^+^ cell population. Data are shown by mean ± SD, *P < 0.05 **; *P* < 0.01; *** *P* < 0.001.

Since Rhodamine B‐labeled H_2_‐PFOB NEs possess fluorescent signal, we first performed ex vivo fluorescence imaging using the IVIS fluorescence imaging system to assess the metabolic distribution of H_2_‐PFOB NEs in normal mice models. The results displayed that the majority of H_2_‐PFOB NEs were metabolized by the liver, spleen and intestine, and cleared from the body after 72 h, whereas a small part of H_2_‐PFOB NEs was metabolized by the respiratory and urinary system (**Figure** **3**a,b). Further, the fluorescent characteristics of I/R group and sham group hearts were also explored by IVIS imaging system, excitingly, the results demonstrated that the excellent targeted accumulation properties of H_2_‐PFOB NEs in I/R myocardium, meanwhile, the fluorescence intensity was also maintained for a prolonged period of time compared with the sham group hearts (Figure 3c,d). This targeted delivery behavior to ischemic myocardium indicates the potential therapeutic advantages of H_2_‐PFOB NEs in treating I/R injury.

To investigate the I/R myocardium targeting mechanisms of H_2_‐PFOB NEs, I/R mice, underwent 60 min of myocardial ischemia, were sampled after 1 h injection of H_2_‐PFOB NEs to explore the cellular uptake properties of H_2_‐PFOB NEs at the early stage of myocardial reperfusion. Corresponding immunofluorescence results indicated that H_2_‐PFOB NEs highly co‐localized with the myocardial necrosis area (Figure 3e). To further examine the cell population containing the H_2_‐PFOB NEs, I/R myocardium tissue sections were incubated with FITC‐labelled anti‐CD11b antibody. As shown in Figure 3f‐g, the fluorescent signal of Rhodamine B‐labelled H_2_‐PFOB NEs overlapped to a large extent with macrophages in the ischemic area and border zone, but not in the non‐ischemic myocardium. TEM was used to observe the detailed distribution of H_2_‐PFOB NEs in the myocardium. A significantly higher number of macrophages was detected in the ischemia area of the myocardium, and a majority of H_2_‐PFOB NEs were engulfed by macrophages via phagocytosis (Figure 3h). After tail vein injection of FITC labeled H_2_‐PFOB NEs and subsequent collection of blood samples, we analyzed different cell populations containing the H_2_‐PFOB NEs. As shown in Figure 3i‐q, 1 h after injection of FITC‐labeled H_2_‐PFOB NEs, cells positive (+) for FITC were detected. The large majority of the labeled cells exhibited the monocyte/macrophage marker CD11b, and only a marginal amount of lymphocytes (CD3) positive for FITC. Furthermore, we found that more than 90% of CD11b positive cells expressing Ly6c (inflammatory monocytes), and which account for 86.6% FITC^+^ CD11b^+^ cells. The above flow cytometric results revealed that H_2_‐PFOB NEs are avidly taken up by inflammatory monocytes in circulating blood. In addition, the flow cytometry analysis of myocardial tissue further delineated that H_2_‐PFOB NEs were mainly obtained by CD11b^+^ F4/80^+^ macrophages. We also notice that CD11b^+^ cells were significantly enhanced in I/R mice, and about half of them positive for FITC, of which more than 90% cells expressed F4/80 macrophages. Most importantly, macrophages account for 97% of FITC^+^ CD11b^+^ cells. Above results fully illustrated that the inflammatory monocytes/macrophages in circulating blood or I/R myocardial tissue mainly contributed to H_2_‐PFOB NEs uptake and distribution. The significant increase in H_2_‐PFOB NEs uptake was also observed in macrophages and cardiomyocytes subjected to LPS‐induced inflammation and hypoxia/reoxygenation (H/R), respectively (Figure S1 and S2, Supporting Information).

**Figure 4:**
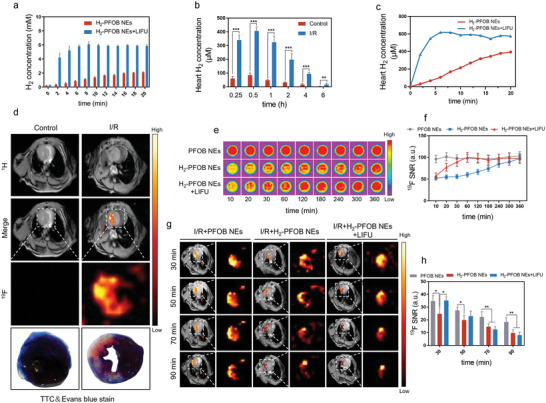
LIFU responsiveness and visualization of hydrogen release of H_2_‐FPOB NEs. a) The concentrations of H_2_ released from H_2_‐Water and H_2_‐PFOB NEs were determined by gas chromatography. b) H_2_ concentration in normal mice and I/R mice were measured by H_2_ microelectrodes at different time after inject of H_2_‐FPOB NEs. c) Hydrogen concentrations were measured in the myocardium of I/R mice with and without LIFU within 20 min after injection of H_2_‐FPOB NEs using H_2_ microelectrodes. d) ^19^F‐MRI of H_2_‐PFOB NEs in normal mice and I/R mice heart (MRI examination at the beginning of reperfusion). e) Representative ^19^F‐MRI phantom images of PFOB NEs, H_2_‐PFOB NEs and H_2_‐PFOB NEs + LIFU at different time points. f) Corresponding ^19^F‐MRI signal intensity calculation of above groups, n = 5. ching cardiac images of I/R mice at different time points after injection (MRI examination at the beginning of reperfusion). h) The ^19^F signal‐to‐noise ratio (SNR) was calculated over time in the myocardium of above groups, n = 5. Data are shown by mean ± SD, *P < 0.05 **; *P* < 0.01; *** *P* < 0.001.

### 2.3 Hydrogen Controlled Release Profiles and ^19^F‐MR Detection Ability

To investigate the hydrogen‐controlled release profiles of H_2_‐PFOB NEs, low intensity focused ultrasound (LIFU) was utilized to excite the nanoparticles. As expected, gas chromatography of H_2_‐PFOB NEs + LIFU or H_2_‐PFOB NEs showed hydrogen burst release only in the H_2_‐PFOB NEs + LIFU group (**Figure** 4a). Next, the targeting ability of H_2_‐PFOB NEs to the I/R myocardium was determined by measuring cardiac hydrogen levels in I/R injury models or normal mice with microelectrodes. Hydrogen concentration in I/R mice hearts peaked at higher values and was sustained for longer than in normal mice hearts (Figure 4b). To further investigate the controlled burst release of hydrogen from H_2_‐PFOB NEs in the I/R myocardium, hydrogen concentration in I/R myocardial tissue combined with LIFU was measured immediately following the injection of H_2_‐PFOB NEs. LIFU rapidly increased hydrogen concentration in the I/R myocardium, exceeding the peak cardiac H_2_ concentration after intravenous H_2_‐PFOB NEs (Figure 4c). These results suggest that combining H_2_‐PFOB NEs with LIFU is an optimal strategy to achieve the controlled blast release of hydrogen molecules.

To further validate the I/R myocardium specific targeting capability of H_2_‐PFOB NEs at the in vivo level, cardiac ^19^F‐MR imaging and ^19^F magnetic resonance spectra were performed after the mice were intravenous injected with H_2_‐PFOB NEs. The schematic diagram of cardiac ^19^F‐MRI imaging is shown in Figure S3 (Supporting Information). Corresponding ^19^F magnetic resonance spectra of H_2_‐PFOB NEs was shown in Figure S4 (Supporting Information). Most importantly, as can be seen from the ^19^F‐MR signal in Figure 4d, compared to normal myocardium, H_2_‐PFOB NEs was highly infiltrated into the left ventricular free wall of ischemia myocardial tissue following ischemia‐reperfusion, in addition, which was highly consistence with the TTC&Evan Blue staining results, while almost no specific ^19^F‐MR signal was observed in the normal myocardium. Similarly, H_2_‐PFOB NEs produce an enhanced ultrasound signal in the left ventricular (LV) cavity after injection (Figure S5, Supporting Information). To determine whether H_2_ affects the fluorine signal of PFOB NEs, phantoms of hydrogenated PFOB NEs with or without LIFU were immediately scanned and ^19^F‐MR images were dynamically acquired at different time points. Intriguingly, hydrogen could lead to quenching of ≈50% of the PFOB NEs ^19^F‐MR signal (Figure 4e). As the hydrogen was released, the signal was recovered over time. As expected, a more rapid restoration was observed in the H_2_‐PFOB NEs + LIFU group (Figure 4f). Next, we assessed whether ^19^F‐MR imaging can detect H_2_‐PFOB NEs hydrogen‐controlled release in a I/R injury mice model in vivo (Figure 4g). The ^19^F‐MR signal in the I/R injury + H_2_‐PFOB NEs group was influenced by hydrogen at every time point, but especially during the first 30 min after reperfusion, which can be attributed to the fluorine signal quenching effect of H_2_‐PFOB NEs. The blasting release of hydrogen from H_2_‐PFOB NEs controlled by LIFU reached a peak value within 20 min, while a ^19^F‐MR signal fluctuation in the I/R + H_2_‐PFOB NEs group occurred ≈30 min. Compared with the I/R + PFOB NEs group, the decrease of fluorine signal was more pronounced in I/R + H_2_‐PFOB NEs + LIFU group at 50 min, 70 min and 90 min after reperfusion (*P*<0.01) (Figure 4h), showing that H_2_‐PFOB NEs have therapeutic potential to regulate inflammatory response in the I/R myocardium. Moreover, these data also indicate that LIFU can significantly accelerate hydrogen release from H_2_‐PFOB NEs.

### 2.4 In Vivo H_2_‐PFOB NEs Myocardial Ischemia‐Reperfusion Injury Therapeutic Effect

**Figure 5:**
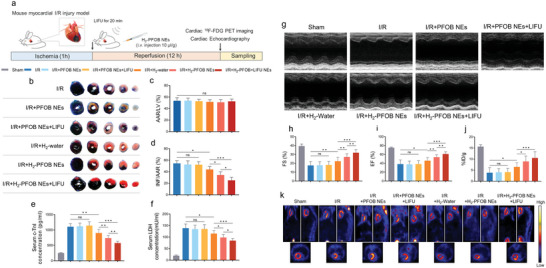
Effects of H_2_‐PFOB NEs on infarct size and cardiac function in I/R mice. a) Schematic illustration of LIFU combined H_2_‐PFOB NEs treatment strategies. b) Representative TTC&Evans blue staining images; c) AAR/LV (%), n = 7. d) INF/AAR (%), n = 7. e) Serum c‐TnI concentrations of each group, n = 7. f Serum LDH concentrations of each group, n = 7. g) Representative echocardiography image of mouse hearts; h) EF (%), n = 15. i) FS (%), n = 15. j) %ID/g, n = 5. k) Representative ^18^F‐FDG PET image of mice's heart. Data are shown by mean ± SD, *P < 0.05 **; *P* < 0.01; *** *P* < 0.001.

Given the hydrogen‐carrying capacity, passive targeting and LIFU‐controlled hydrogen releasing ability of H_2_‐PFOB NEs, we tested the hypothesis that H_2_‐PFOB NEs may exert favorable therapeutic effects on myocardial I/R injury on mice models (**Figure** **5**a). TTC and Evans blue staining showed no significant differences in the left ventricle area at risk (AAR/LV) between any of the groups (Figure 5b,c). However, infarct area ratio (IFN/AAR) results showed that myocardial injury was significantly reduced in the H_2_‐PFOB group when compared to the H_2_‐water group (Figure 5d). When further combined with LIFU (H_2_‐PFOB + LIFU), myocardial death in risk areas was almost completely inhibited when compared with H_2_‐PFOB (*P*<0.05). Cardiac biomarkers were then analyzed by ELISA in the myocardial I/R injury model to evaluate the degree of myocardial damage. While a reduction in cardiac troponin I (c‐TnI) and lactate dehydrogenase (LDH) levels was detected in the saturated H_2_‐Water group (*P*<0.05), in the H_2_‐PFOB and H_2_‐PFOB + LIFU groups the myocardial injury index was nearly completely reversed (Figure 5e,f).

To further explore the effects of H_2_‐PFOB NEs on cardiac function, we conducted cardiac ultrasound on myocardial I/R injury mice (Figure 5g–i). Cardiac contractile function measured by ejection fraction (EF) and fractional shortening (FS) indexes decreased significantly in the H_2_‐PFOB NEs group (EF≈39%, FS≈54%) and to a lesser extent in the H_2_‐water group (EF≈21%, FS≈28%). Notably, when H_2_‐PFOB NEs were combined with LIFU, cardiac contractile function was restored to a greater extent than in the other groups (EF≈60%, FS≈76%). ^18^F‐FDG PET imaging were performed to evaluate the therapeutic efficacy of each treatment groups on I/R myocardium, the results of %ID/g further demonstrated that H_2_‐PFOB NEs have a stronger therapeutic effect in alleviating myocardial damage and improving cardiac function than H_2_‐water, especially when combined with LIFU (Figure 5j,k).

### 2.5 H_2_‐PFOB NEs Myocardial Ischemia‐Reperfusion Injury Therapy Mechanisms

**Figure 6:**
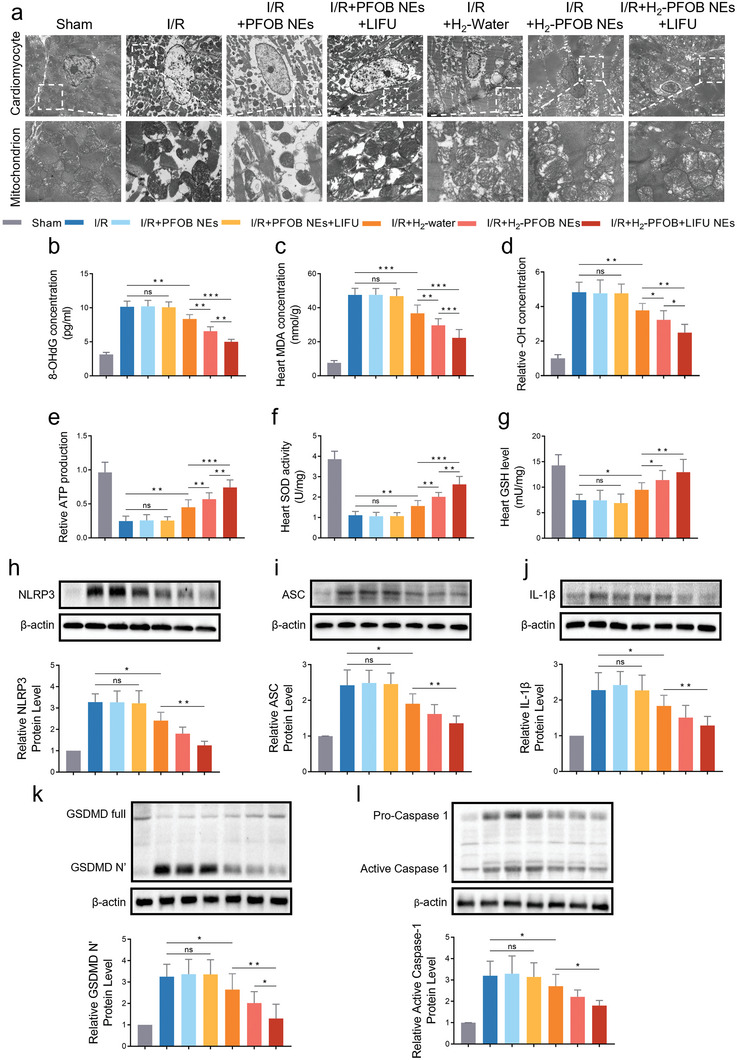
Effects of H_2_‐PFOB NEs on mitochondrial oxidative stress and NLRP3‐mediated pyroptosis in I/R mice. a) TEM images illustrating the cardiomyocytes (×10k, scale bar 100 nm). b) 8‐OHdG concentrations of each group, n = 7. c) Heart MDA concentrations of each group, n = 7. d) Relative ·OH concentrations of each group, n = 7 e) Relative ATP production of each group, n = 7. f Heart SOD activity of each group, n = 7. g) Heart GSH concentrations of each group, n = 7. h) Relative NLRP3 protein level, n = 5. i) Relative ASC protein level, n = 5. j) Relative IL‐1β protein level, n = 5. k) Relative GSDMD protein level, n = 5. l) Relative Caspase‐1 protein level, n = 5 Data are shown by mean ± SD, *P < 0.05 **; *P* < 0.01; *** *P* < 0.001.

Next, we explored the mechanisms underlying the H_2_‐PFOB NEs therapeutic effects described above. TEM reduced numbers of mitochondria and altered mitochondrial morphology, including increased size and disorganized cristae, suggesting that myocardial I/R injury causes mitochondrial oxidative stress (**Figure** **6**a). Importantly, mitochondrial damage in myocardial ischemia was significantly attenuated in the H_2_‐PFOB and H_2_‐PFOB + LIFU groups. Moreover, the levels of the oxidative stress markers 8‐OHdG and malondialdehyde (MDA) decreased significantly the H_2_‐PFOB group, particularly in combination with LIFU (Figure 6b,c). Similarly, measurements of hydroxyl radical (OH) concentrations reveled that H_2_‐PFOB NEs, and H_2_‐PFOB NEs + LIFU have better antioxidant properties than H_2_‐water (Figure 6d). Finally, changes in the mitochondrial damage indicator ATP and oxidative stress markers superoxide dismutase (SOD) and glutathione (GSH), further revealed that mitochondrial oxidative stress in myocardial I/R injury is significantly reduced by H_2_‐PFOB NEs in combination with LIFU (Figure 6e–g).

To further examine the therapeutic effects of H_2_‐PFOB NEs, we analyzed multiple markers of NLRP3‐mediated pyroptosis in myocardial I/R injury models. NLRP3 immunostainings showed that H_2_‐PFOB NEs inhibit the expression of NLRP3 protein in myocardial tissue, and LIFU further enhanced this effect (Figure S6, Supporting Information). Moreover, western blot assays revealed that H_2_‐PFOB NEs + LIFU significantly reduces the expression of the pyroptosis‐related proteins ASC, Caspase‐1, Gasdermin D and IL‐1β in myocardial I/R injury mice models (Figure 6h–l), suggesting that H_2_‐PFOB NEs have a strong anti‐inflammatory effect on the NLRP3 inflammasome. Together these results strongly suggest that H_2_‐PFOB NEs have important therapeutic effects on myocardial I/R injury by reducing oxidative stress and decreasing NLRP3 inflammasome activation‐mediated pyroptosis.

### 2.6 In Vivo H_2_‐PFOB NEs Myocardial Ischemia‐Reperfusion Injury Prognostic Effect

**Figure 7:**
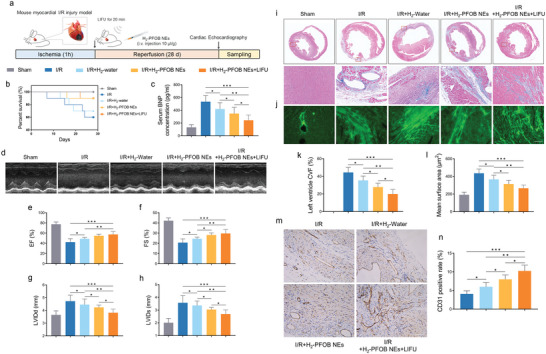
Effects of H_2_‐PFOB NEs on long term cardiac structure and myocardial fibrosis in I/R mice. a) Schematic illustration of LIFU combined H_2_‐PFOB NEs treatment strategies. b) Survival curve of rats of each experimental group. c) The Serum BNP concentration of each group, n = 5; d) Representative echocardiography image of mice's heart. e–h) EF (%); FS (%); LVIDd and LVIDs, n = 7; i) The representative Masson images of mice heart; j) The representative immunohistochemical staining images of wheat germ agglutinin (WGA) expression in each group (×20, scale bar 50 µm). k) Collagen volume fraction (CVF) of left ventricle, n = 5; l) Quantitative analysis of left ventricular tissue sections stained with WGA, n = 5; m,n) The representative immunohistochemical staining images of CD31 and quantitative analysis of CD31 positive rate, n = 5 (×20, scale bar 50 µm). Data are shown by mean ± SD, *P < 0.05 **; *P* < 0.01; *** *P* < 0.001.

Patients with ischemic reperfusion injury have a high probability of long‐term cardiac remodeling and heart failure, which seriously affect the quality of life and survival of patients. Therefore, based on the favorable therapeutic effects of H_2_‐PFOB NEs during the acute phase of I/R injury, we further evaluated the long‐term outcomes of I/R (1 h of ischemia and 28 days of reperfusion) mouse treated with H_2_‐PFOB NEs. The study schema is shown in **Figure** **7**a, corresponding results were powered to show a reduction in mortality of I/R mouse treated with H_2_‐PFOB NEs (Figure 7b). Compared with H_2_‐water group, H_2_‐PFOB and H_2_‐PFOB + LIFU groups could further reduce the concentration of myocardial injury index BNP in serum (*P*<0.05) (Figure 7c). Transthoracic echocardiography was used to assess cardiac structure and function of mice in different treatment groups. Improvement in left ventricular ejection fraction (EF) occurs in H_2_‐PFOB + LIFU group when compared with other groups (57.4% versus 54.7% versus 48.8%). In addition, the left ventricular fractional shortening (FS) was measured and the results demonstrated that H_2_‐PFOB and H_2_‐PFOB + LIFU groups are better able to improve systolic function in infarcted mouse hearts than H_2_‐Water group. The measurement results of left ventricular end‐diastolic internal diameter (LVIDd) and left ventricular end‐systolic internal diameter (LVIDs) were illustrated that H_2_‐PFOB and H_2_‐PFOB + LIFU groups could better prevent the onset of the subsequent deterioration of long‐term cardiac function and cardiac remodeling in I/R mice (Figure 7d–h). Furthermore, to assess the cardiac remodeling and myocardial fibrosis, paraffin‐embedded heart sections were stained with Masson trichrome staining. As expected, H_2_‐PFOB NEs significantly reduced myocardial fibrosis, especially in the H_2_‐PFOB + LIFU group (Figure 7i,k). WGA and CD31 immunohistochemical staining results showed that H_2_‐PFOB NEs could moderate hypertrophy of surviving cardiomyocytes and promote the development of neovascularization, which further contributes to the prevention of the long‐term heart failure and cardiac remodeling caused by I/R injury (Figure 7j–n).

### 2.7 Cytotoxicity and Biosafety of H_2_‐PFOB NEs

H_2_‐PFOB NEs cytotoxicity was assessed in primary cardiomyocytes using standard CCK‐8 cell viability assays. H_2_‐PFOB NEs exhibited negligible cytotoxicity in primary cardiomyocytes even at high concentrations (Figure S7, Supporting Information). Moreover, no obvious body weight loss was observed in balb/c mice treated with H_2_‐PFOB NEs (Figure S8, Supporting Information). In vivo toxicity of H_2_‐PFOB NEs was assessed by histological analyses of major organs (brain, heart, liver, spleen, lung, and kidneys) harvested 24 h after H_2_‐PFOB NEs NEs injection. As shown in Figure S9 (Supporting Information), H_2_‐PFOB NEs dosed at 10 µL g^−1^ 30% (v/v) induced no obvious pathological changes, including on cytoplasm loss, cell atrophy or inflammation, further suggesting that these nanoemulsions have excellent histocompatibility.

In addition, standard biochemical analyses revealed no changes in blood urea (UREA) or creatinine (CREA) levels in the H_2_‐PFOB NEs group, indicating normal kidney function. Alanine aminotransferase (ALT), alkaline phosphatase (ALP), and aspartate aminotransferase (AST) levels also remained normal in the H_2_‐PFOB group at days 1 and 7 after injection, demonstrating healthy liver function. Finally, no significant changes were detected in red blood cells (RBCs), mean corpuscular hemoglobin (MCH), white blood cells (WBCs), mean corpuscular hemoglobin concentration (MCHC), platelet (PLT), hemoglobin (HGB), mean corpuscular volume (MCV), hematocrit (HCT), mean platelet volume (MPV), or platelet distribution width (PDW), showing that mice injected with H_2_‐PFOB NEs have no apparent infections or defects in the physiological regulation of their immune system (Figure S10, Supporting Information).

## 3 Discussion

Myocardial ischemia‐reperfusion (I/R) injury has an important negative impact on the therapeutic effects of revascularization therapy, thereby challenging the development of effective therapeutic drugs.^[^ ^14^ ^]^ The deleterious role of reactive oxidative stress (ROS) on cardiac muscle has been comprehensively studied in I/R injuries, and it is well established that a burst of mitochondrial‐derived ROS contributes to muscle damage.^[^ ^15^ ^]^ Moreover, the involvement of ROS in oxidative stress‐induced myocardial dysfunction is particularly relevant to the development of targeted therapies.^[^ ^16^ ^]^ Hydrogen has a protective ability against oxidative stress and inflammation, and extensive research has demonstrated the preclinical and clinical efficacy of hydrogen in the treatment of a variety of inflammatory‐associated diseases, including diabetes, atherosclerosis and cancer.^[^ ^4^, ^17^ ^]^ In a previous study, our research has shown that hydrogen can reduce myocardial I/R injury by inhibiting ROS‐mediated pyroptosis. Moreover, hydrogen also plays a major role in reducing the no‐reflow phenomenon and in alleviating cardiac remodeling and myocardial fibrosis after myocardial infarction.^[^ ^18^ ^]^ However, current hydrogen delivery strategies release low amounts of uncharged hydrogen molecules that can quickly enter the cytoplasm, mitochondria and even the nucleus,^[^ ^19^ ^]^ which affects hydrogen dosage and therefore reduces therapeutic efficacy.^[^ ^8^ ^]^ Thus, the development of more advanced methods for targeted delivery and controlled release of hydrogen is critical to improve hydrogen‐based therapy.

PFCs, a group of chemically inert synthetic molecules with excellent biocompatibility, have been widely studied and used in the clinic for various purposes, including artificial blood substitution, organ preservation, ultrasound imaging, and fluorine magnetic imaging.^[^ ^11^ ^]^ The major advantage of PFCs stems from their unique ability to dissolve significant amounts of oxygen, owing to van der Waals forces.^[^ ^20^ ^]^ To our knowledge, this is the first study to explore the hydrogen‐carrying capacity of PFOB NEs. We have successfully developed PFOB NEs with high hydrogen‐carrying capacity, LIFU‐controlled release, and optimal biocompatibility. PFOB NEs offer multiple advantages, such as ischemic myocardium‐specific targeting and ^19^F‐MR‐based dynamic visualizing of hydrogen release. In contrast to other hydrogen carriers,^[^ ^21^ ^]^ these properties make H_2_‐PFOB NEs a promising candidate in precision medicine for the treatment of various diseases. Indeed, given their excellent biosafety profile, H_2_‐PFOB NEs have significantly better clinical translational potential than current hydrogen carriers.^[^ ^22^ ^]^

Since myocardial I/R injury mediated by oxidative stress and inflammation is more evident in the early phase of the injury process,^[^ ^23^ ^]^ we administrated H_2_‐PFOB NEs at the beginning of the reperfusion period. H_2_‐PFOB NEs can rapidly infiltrate into the targeted site and release hydrogen efficiently, particularly when used in combination with LIFU. Hydrogen concentration in ischemic area reaches a peak at the early stage of reperfusion, thereby effectively alleviating oxidative stress damage and inflammasome activation of I/R injury, which in turn reduces infarct size and restores cardiac function. Thus, H_2_‐PFOB NEs used in combination with LIFU maximize protection against myocardial I/R injury.

Previously developed hydrogen‐loaded nanocarriers have therapeutic applications mostly for the treatment of cancer and osteoarthritis.^[^ ^24^ ^]^ In this study, we report a novel hydrogen carrier, H_2_‐PFOB NEs, and describe its therapeutic properties and molecular mechanisms of myocardial I/R injury recovery in preclinical models. Thus, we have uncovered a new ^19^F‐MR imaging‐guided strategy for the diagnosis and precise treatment of myocardial I/R injury and expanded the scope of clinical applications of hydrogen carriers. With exciting translational prospects ahead, future large animal in vivo investigations are necessary to further assess the role of H_2_‐PFOB NEs in I/R myocardium precision therapy, moreover, the potential therapeutic benefits of H_2_‐PFOB NEs in other diseases certainly deserves further investigation.

## 4 Conclusion

In this study, we developed an advanced precise treatment strategy for myocardial I/R injury with potential clinical applications in other diseases. H_2_‐PFOB NEs have high hydrogen load capability and can passively infiltrate into ischemic myocardial tissue via a macrophage‐mediated process. In combination with LIFU, H_2_‐PFOB NEs exert remarkable therapeutic effects on I/R injury due to the LIFU‐mediated controlled release of hydrogen in targeted injury areas. Moreover, to our knowledge H_2_‐PFOB NEs is the first ^19^F‐MRI molecular imaging probe that allows the dynamic visualization of hydrogen release in vivo. Finally, our data suggest that H_2_‐PFOB NEs have excellent antioxidant stress and anti‐inflammatory properties which further contribute to the reversal of I/R injury. Thus, H_2_‐PFOB NEs in combination with LIFU is a novel hydrogen delivery therapeutic agent with great potential for clinical translation.

## 5 Experimental Section

### Animals

Balb/c mice weighing 22–25 g were obtained from Charles River Labs (Beijing, China). The animals were bred and housed under standard conditions (12 h light‐dark cycle, 24 °C), with free access to water and standard laboratory chow. The animal experimental protocol was approved by the Institutional Animal Care and Use Committee of Harbin Medical University (Approval No. 2021WZYSLLSC‐20).

### Chemicals and Preparation of Perfluorocarbon Nanoemulsions (NEs)

Unless otherwise listed, all solvents and reagents were purchased from Aldrich Chemical Co. and used as received. Phospholipids were purchased from Avanti Polar Lipids, Inc. (Alabama, USA). Perfluorooctylbromide (PFOB, C_8_BrF_17_) was purchased from Exfluor Research Corporation (Round Rock, TX) and used as acquired.

The fluorescently tagged perfluorocarbon nanoemulsions (NEs) were synthesized using microfluidization similar to prior reports.^[^ ^25^ ^]^ Briefly, the nanoparticulate emulsions were comprised of 30% (v/v) PFOB, 3% (w/v) of a surfactant commixture. The surfactant included 80 mol% dipalmitoyl phosphatidylcholine (DPPC), 0.5 mol% 1,2‐dipalmitoyl‐sn‐glycero‐3‐phosphoethanolamine (DPPE), 0.2 mol% dipalmotoylphosphatidylglycerol (DPPG), 19.3 mol% cholesterol, 0.02 mol% lissaminerhodamine B sulfonyl (16:0 LissRhod PE). The lipids were dissolved in a mixture of methanol and chloroform, filtered through a small bed of cotton, evaporated under reduced pressure using a rotary evaporator at 45 °C to form a thin film, and then further dried in a vacuum oven (45 °C) for 24 h. The resuspended surfactant was combined with PFOB and water, and then emulsified in a M110P Microfluidics emulsifier (Microfluidics, Newton, MA) at 20,000 psi for 4 min. The completed emulsions were placed in crimp‐sealed vials, blanketed with argon, and stored at 4 °C until use.

### Physical and Chemical Characterization of the H_2_‐PFOB NEs

Hydrodynamic diameter distribution, polydispersity, and zeta potential (ζ) of the PFC nanoparticles were determined by dynamic light scattering (DLS) with a Malvern Nano ZS Zetasizer (Malvern Instruments Ltd, Malvern, UK). All determinations were made in multiples of three consecutive measurements. The absorbance of Rhodamine B was measured by using a multiplate reader using the option UV Absorbance Spectrophotometer (BioTek, Winooski, VT).

The morphology and microstructure of the PFOB NEs were determined by transmission electron microscopy (TEM) on a Hitachi‐7700 microscope at an accelerating voltage of 220 kV. Briefly, a drop of the sample was deposited on a carbon grid coated with copper and the excess sample was drawn off with filter paper 1 min later and was then left for 5 min to be dried. Digital TEM photographs of the PFOB NEs were obtained.

### Measurement of Hydrogen Release Behavior of the H_2_‐PFOB NEs

The amount of dissolved hydrogen (H_2_) and the release rate of H_2_ in pure water and perfluorocarbon (30 % v/v) solution were measured with a gas chromatograph and a hydrogen microelectrode. Specifically, saturated hydrogen water was diluted with pure water in different proportions to prepare a standard curve. Next, hydrogen concentrations in H_2_‐PFOB NEs, and hydrogen concentrations in the heart after H_2_‐PFOB NEs injection were measured.

### Metabolic Distribution In Vivo

In vivo degradation of the nanoemulsions was monitored with an In Vivo Imaging System (IVIS Lumina XR, USA) after Balb/c mice were intravenously injected with H_2_‐PFOB NEs (10 µL g^−1^, 30% (v/v) PFOB NEs). The mice were sacrificed 1 h, 12 h, 24 h, and 72 h after injection, and all major organs (heart, liver, spleen, lung, kidney, intestine, muscle, and bone) were harvested and imaged ex vivo using an IVIS Spectrum. The average total fluorescence intensity of each organ was semi‐quantified using the IVIS imaging software.

### Animal 9.4 T Magnetic Resonance Imaging (MRI) Experiments

All magnetic resonance imaging (MRI) experiments were performed on a Bruker Biospin 9.4T scanner (Bruker BioSpin 94/20 USR system, Germany) with a ^1^H/^19^F double tune volume coil. After acquisition of the morphological ^1^H images, the resonator was tuned to ^19^F and the corresponding ^19^F‐MR images were recorded from the same FOV using a ^19^F chemical shift‐selective (CSSI) pulse sequences with multislice rapid acquisition. Mice were anaesthetized by inhalation with an initial dose of 2 % isoflurane (RWD Life Science) and were maintained spontaneously breathing 1.5% isoflurane, supplied via a nose cone. During imaging, respiratory rates were continuously monitored by a small animal monitoring and gating system (SA Instruments), and the body temperature was maintained at 37 °C using a temperature‐controlled heating system (Thermo). Parameters of ^19^F_CSSI sequence: RARE factor, 64; matrix, 64×64; slice thickness, 3 mm; averages, 128; acquisition time, 8.32 minutes. For fusion with ^19^F images, additional ^1^H data sets with a slice thickness of 1.0 mm were recorded.

### Low‐Intensity Focused Ultrasound (LIFU)

The parameters and settings of low‐intensity focused ultrasound (LIFU, Shenzhen Institute of Advanced Technology, Chinese Academy of Sciences) were as follows: Focus area of 0.4 cm^2^, pulse wave mode, 50 % duty cycle, 1.1 MHz, 55.8 mW cm^−2^, and focal length of 2 cm. LIFU acoustic intensity was confirmed by hydrophone. The schematic diagram of experimental procedure of LIFU H_2_‐PFOB NEs therapy applied to myocardial ischemia‐reperfusion injury and animal grouping were shown in Figure 6a. Specifically, H_2_‐PFOB NEs (10 µL g^−1^, 30% (v/v)) were administered to mice 1 h after ischemia that exactly at immediate reperfusion the infusion was done and followed by LIFU irradiation for 20 min to facilitate controlled release of the hydrogen at the targeted area.

### Mouse Myocardial Ischemia‐Reperfusion Model

Balb/c mice were anesthetized with Ketamine/Xylazine mixture, intubated, and ventilated with a Ventstar mouse ventilator (RWD; stroke volume, 250 µL; respiratory rate, 105 breaths per minute). An ECG device was used for electrocardiogram (ECG) monitoring of mice in real time to ensure the experimental model was suitable. Following left thoracotomy between the fourth and fifth ribs, a 7‐0 nylon suture was placed around the left coronary artery for ligation, with the aid of a dissecting microscope. The ischemic state of heart was confirmed by evidence of immediate visible changes, including sudden pallor and paralysis of the affected part of the left ventricle, accompanied by an ST‐T segment elevation detected in the ECG. After 60 min of ischemia, the ligature was then released, the chest was closed in layers and the mice were allowed to recover^[^ ^3a^ ^]^. Next, H_2_‐Water, PFOB NEs and H_2_‐PFOB NEs (10 µL g^−1^, 30% (v/v) PFOB) were injected through the caudal vein. The dosage of PFOB injected refers to previous published studies^[^ ^26^ ^]^. In the sham group, the chest was opened and the suture needle passed through left coronary artery, but it was not ligated.

### Infarct Size Measurement

After reperfusion, each mouse group was anesthetized with a Ketamine/Xylazine complex and a thoracotomy was performed. Then, the left ventricle (LV) areas at risk (AAR) were assessed for each mice group with 1 ml of 2 % Evan's blue dye (Sigma, E2129, USA) perfusion while ensuring that the left coronary artery (LCA) was re‐occluded at the same position. Next, the hearts stained with Evan's blue were removed rapidly and frozen at −20 °C for 15 min after sacrifice and then sliced horizontally at 1–2 mm thickness under the ligation line, to compare the myocardial tissues of the different groups. The heart slices were immersed in 1 % 2,3,5‐triphenyltetrazolium chloride (TTC) (Sigma, T8877, USA) phosphate buffer saline (PBS) solution for 15 min at 37 °C. The viable myocardial tissue was displayed in red and the nonviable myocardial tissue appeared to be white. The stained slices were imaged with a digital camera, and the total LV area, the AAR, and the infarct area were quantified using an Image‐Pro Plus software. AAR was expressed as a percentage of the LV (AAR/LV), and infarction (INF) per area at risk was expressed as a percent of the AAR (INF/AAR).

### Echocardiographic Assessment of Cardiac Function

Cardiac function was evaluated by 2D transthoracic echocardiography on conscious mice using a VisualSonics Vevo2100 imaging system, as described previously^[^ ^27^ ^]^. Fractional shortening (FS) and ejection fraction (EF) were used as indices of cardiac contractile function. FS was calculated according to the following formula: FS = (LVIDd‐LVIDs)/LVIDd × 100%. EF was calculated as EF = [(LVIDd)^3^‐(LVIDs)^3^]/(LVIDd)^3^ × 100%. All measurements were performed by an experienced operator blinded to the study.

### Heart ^18^F‐FDG Positron Emission Tomography (PET) Imaging

^18^F‐FDG was obtained as an aliquot from the daily production for the clinical positron emission tomography (PET) at the Fourth Affiliated Hospital of Harbin Medical University qualified for administration in humans. ≈0.15≈0.2 mCi of ^18^F‐FDG per mouse was administered intravenously. Mice micro‐PET imaging was acquired 45 min after ^18^F‐FDG injection using a Super Argus PET scanner (Sedecal, Spain). ROI of myocardial ischemia was outlined by Amide software, and FDG uptake of ischemic myocardium was quantitatively analyzed by % ID/g, as previously reported.^[^ ^28^ ^]^

### ELISA

Final blood was collected in 1% EDTA, centrifuged at 3000 rpm for 15 min and the plasma collected. The plasma concentrations of lactate dehydrogenase (LDH) (Cusabio Bio, CSB‐E11723m, China), cardiac troponin I (c‐TnI) (Cusabio Bio, CSB‐E08421m, China) and 8‐hydroxy‐2 deoxyguanosine (8‐OHdG) (Cusabio Bio, CSB‐E10527m, China) were measured using ELISA kits, according to the manufacturer's instructions.

### Measurement of Malondialdehyde and Hydroxyl Radical Concentrations

Malondialdehyde (MDA) concentration was measured with a commercial kit (Jiancheng BioEngineering Institute, A003‐2‐1, China) to monitor oxidant‐mediated lipid peroxidation. The concentration of hydroxyl radical (OH), a type of toxic ROS, was measured with commercial kit (Jiancheng BioEngineering Institute, A018‐1‐1, China).

### Measurement of ATP, Superoxide Dismutase (SOD) and Glutathione (GSH) Level

ATP concentration was measured with a commercial kit (Beyotime, S0026, China) to monitor mitochondrial function. Oxidative stress balance index GSH (Solarbio Life Science, BC1175, China) and SOD concentration (Solarbio Life Science, BC0170, China) were measured by using a commercial kit.

### Flow Cytometry

Flow cytometry were conducted to further explore the underlying mechanism of the selective accumulation of H_2_‐PFOB NEs in the injured heart. I/R mice were injected with 10 µL g^−1^ FITC labeled probe, respectively, at 1 h post injury. 30 min after the injection, the blood of the mice was stained with specific antibodies, APC anti‐mouse CD45 (#103111, Biolegend, USA), PE/Cyanine7 anti‐mouse CD3 (#100219, Biolegend, USA), APC/Cyanine7 anti‐mouse CD11b (#101225, Biolegend, USA), PE anti‐mouse Ly‐6C (#128007, Biolegend, USA) and PE anti‐mouse F4/80 (#123109, Biolegend, USA). After centrifugation at 1000 rpm for 5 min, the cells were resuspended in Staining Buffer, and fluorescence signals were detected by BD FACS Aria III. None‐cardiomyocytes single‐cell suspension of tissues in the marginal area of the injury was obtained by using Multi Tissue Dissociation Kit 2 (MiltenyiBiotec, USA) according to the instructions. Briefly, after cut into small pieces (1–2 mm^3^), harvested heart tissue was digested in Enzyme Mix at 37 °C for 15 min, followed by mechanical agitation through the Program Multi_G of the gentle MACS Dissociator (MiltenyiBiotec, USA), and then repeated once. After filtered through MACS SmartStrainer (70 um), the resulting sample was centrifuged at 600 g for 5 min to separate singe cells. The cell pellet of one sample was resuspended in 10 µL g^−1^ staining buffer. Follow‐up staining and detection process were the same as described previously.

### Western Blotting Analysis

Protein samples were extracted using lysis buffer containing 1 % protease inhibitor (Roche, Switzerland). Protein concentrations were measured using a BCA protein kit (Beyotime Institute of Biotechnology, Shanghai, China). The proteins were separated by 12% sodium dodecyl sulfate‐polyacrylamide gel electrophoresis (SDS) and transferred onto polyvinylidene difluoride membranes (PALL, USA). The membranes were incubated with primary antibodies against NLRP3 (NBP2‐12446, Novus, USA; 1:200), GSDMD (sc‐393581, Santa Cruz Biotechnology, USA; 1:200), IL‐1β (ab9722, Abcam, USA; 1:1000), caspase‐1 (22915–1‐AP, Santa Cruz Biotechnology, China; 1:200), ASC (sc‐514414, Santa Cruz Biotechnology, USA1:200) and β‐actin (1:1000; #bs‐0061R, BIOSS, Boston, MA, USA) at 4 °C overnight. After washing with T‐BST, the membranes were inoculated at room temperature with horseradish peroxidase‐labelled secondary antibodies (SA00001‐1 and 2, Proteintech, China; 1:2000) for one hour. Next, ECL reagent was employed to develop the bands and the protein bands were detected by autoradiography and analyzed with ImageJ software. β‐actin was used as a normalized reference.

### Masson and Wheat Germ Agglutinin (WGA) Stain

Hearts were harvested, fixed with 4% paraformaldehyde, and then paraffin‐embedded. After that, 5 mm sections were made. Masson staining were employed to assess cardiac fibrosis along with collagen deposition by a Masson stain kit. The whole heart fibrosis (fibrosis area/whole heart area) was computed as the ratio of fibrosis area to the overall or left ventricular assessed area quantified with the Image J software (V.1.50, Bethesda, USA). The sections of hearts were prepared and stained with WGA following the instruction of manufacture protocol. The green fluorescence indicates the cell membrane, and the surface area of cardiomyocyte was measured by Image J software.

### Statistical Analysis

All data were expressed as mean ± SD. Data normality was determined by Shapiro‐Wilk test. Student's t‐test was used for comparisons between two groups. One‐way ANOVA followed by Bonferroni post hoc analysis was used when comparing > 2 groups. Two‐way ANOVA followed by Bonferroni post hoc analysis was used when more than 2 groups and variables were compared. *P*<0.05 was considered statistically significant. Prism 9.0 (GraphPad Inc, San Diego) and IBM SPSS Statistics 22.0 (IBM Co., Armonk, New York) were used for the statistical analyses.

## Conflict of Interest

The authors declare no conflict of Interest.

## Author Contributions

C.N., R.A, and J.W. contributed equally to this work. C.N. and X.S. designed the experiment and drafted the manuscript. X.S. supervised whole study and provided invaluable advice and assistance. W.Y. and X.S. developed the research idea. L.W provides PFOB NEs. C.N. and R.A. performed the statistical analyses, as well as interpreted the results and drafted the manuscript. J.W completed the cardiac ^18^F‐FDG PET imaging of mice. S.P., R.Z., B.W., S.X., X.H., M.Z., H.W., M.Y. helped with the experiments. The authors read and approved the final manuscript.

## Supporting information

Supporting Information

## Data Availability

The data that support the findings of this study are available from the corresponding author upon reasonable request.
