# Supplementary material for: Controlled Release of Hydrogen‐Carrying Perfluorocarbons for Ischemia Myocardium‐Targeting 19F MRI‐Guided Reperfusion Injury Therapy
Source: Adv Sci (Weinh). 2023 Aug 18;10(29):2304178. doi: 10.1002/advs.202304178 (PMC10582447; doi:10.1002/advs.202304178)
Supplement: Supplementary file 1 — Supporting Information [file ADVS-10-2304178-s001.pdf]

ADVANCED SCIENCE
Open Access

Supporting Information

for *Adv. Sci.*, DOI 10.1002/advs.202304178

Controlled Release of Hydrogen-Carrying Perfluorocarbons for Ischemia Myocardium-Targeting ^{19}F MRI-Guided Reperfusion Injury Therapy

Chaoqun Nie, Rong A, Jing Wang, Shuang Pan, Rentong Zou, Bin Wang, Shuiqing Xi, Xiaojian Hong, Meifang Zhou, Haoyu Wang, Mengshu Yu, Lina Wu, Xilin Sun* and Wei Yang**

Supporting Information

Controlled Release of Hydrogen-carrying Perfluorocarbons for Ischemia Myocardium-targeting ^{19}F MRI-guided Hydrogen Reperfusion Injury Therapy

Chaoqun Nie[1,4#], Rong A[2,3#], Jing Wang[2,3#], Shuang Pan[1], Rentong Zou[1], Bin Wang[1], Shuiqing Xi[1], Xiaojian Hong[1], Meifang Zhou[2,3], Haoyu Wang[2,3], Mengshu Yu[1], Lina Wu[2,3]*, Xilin Sun[2,3]*, Wei Yang[1]*

[1] Department of Cardiology, the Fourth Hospital of Harbin Medical University, Harbin 150000, China

[2] Department of Nuclear Medicine, the Fourth Hospital of Harbin Medical University, Harbin 150000, China

[3] NHC Key Laboratory of Molecular Probe and Targeted Theranostics, Molecular Imaging Research Center (MIRC) of Harbin Medical University, Harbin 150000, China

[4] Department of Cardiology, Laboratory of Heart Center, Zhujiang Hospital, Southern Medical University, Guangzhou 510000, China

[#] These author have contributed equally to this work

* Correspondence:

Wei Yang, M. D. Department of Cardiology, the Fourth Hospital of Harbin Medical University. 37 Yiyuan Street, Nangang District, Harbin, Heilongjiang, P.R. China, 150001. Office: +86-451-86669102. E-mail: hydyangwei@tom.com

Xilin Sun, M. D. Department of Nuclear Medicine, the Fourth Hospital of Harbin Medical University. 766 Xiangan North Street, Songbei District, Harbin, Heilongjiang, P.R. China, 150028. Office: +86-451-88118621. E-mail: sunxl@ems.hrbmu.edu.cn

Lina Wu, M. D. Department of Nuclear Medicine, the Fourth Hospital of Harbin Medical University. 766 Xiangan North Street, Songbei District, Harbin, Heilongjiang, P.R. China,

150028. Office: +86-451-88118621. E-mail: LinaWu@hrbmu.edu.cn

Methods

In vitro experiment protocol

Mouse macrophage cell line (Raw 264.7 cells) and myocardial cell line (H9C2 cells) were cultured in a cell incubator containing 5% CO_2 at 37 °C, and the in vitro experiment was started when the cells in the culture dish were fused to 80%. The inflammatory activation of macrophages was triggered by adding 100ng/ml lipopolysaccharide (Sigma, USA) and incubated for 24h. The hypoxia/reoxygenation (H/R) model of cardiomyocytes was achieved in a self-made hypoxia incubator (hypoxia 1h/ reoxygenation 1h). Then, 20 μl H_2-PFOB NEs were added to the cell culture medium and incubated with the cells for 1 h, 2 h and 4 h, respectively.

In vivo biosafety experimental protocol

Balb/c mice were selected for routine blood tests after intravenously injected with PFOB NEs or H_2-PFOB NEs (200 μL, 30% (v/v). The control group mice were injected with the same dose of saline. The weight changes of the mice were daily monitored. The blood samples were collected on day 1 and day 7 post H_2-PFOB NEs injection, and analyzed with a hematology analyzer. Major organs (heart, liver, spleen, lungs, kidneys) were colleted for HE staining.

Figure

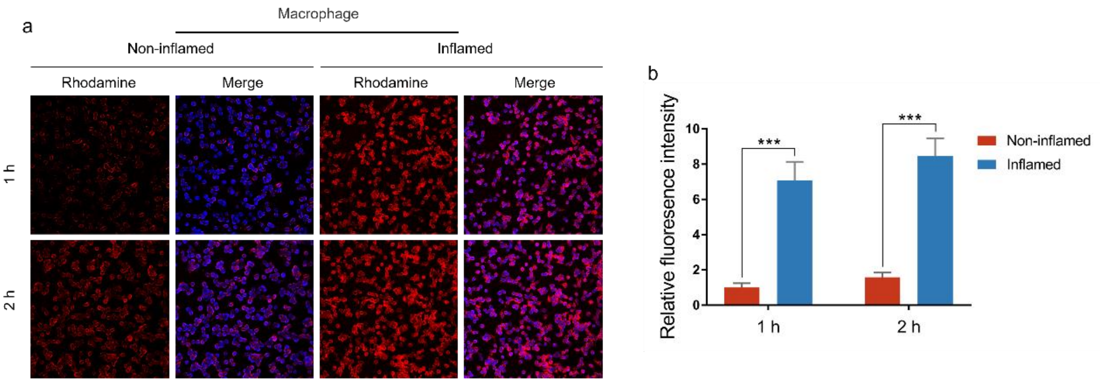

Supplementary Fig. 1. Representative immunofluorescence images of Rhodamine labeled H_2-PFOB NEs incubated with inflamed or non-inflamed macrophages for different time. b

Quantitative analysis.

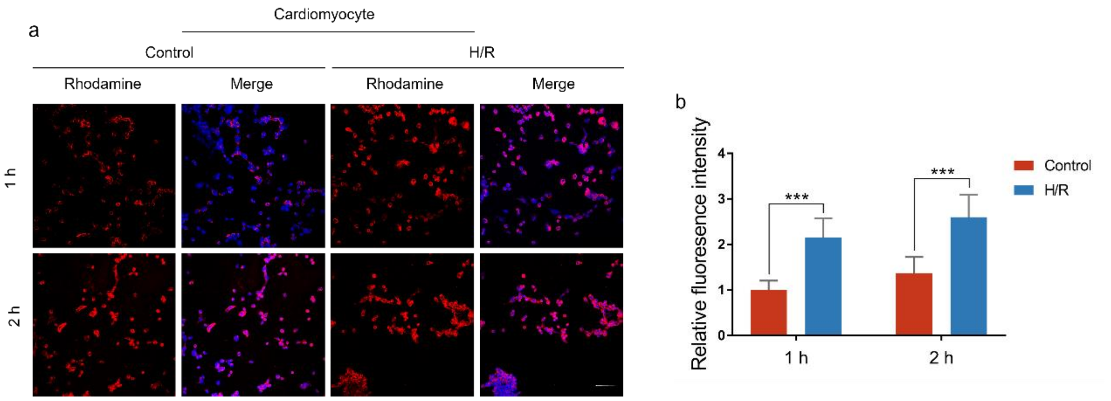

Supplementary Fig. 2. a Representative immunofluorescence images of Rhodamine labeled H_2-PFOB NEs incubated with control or H/R cardiomyocyte. b Quantitative analysis.

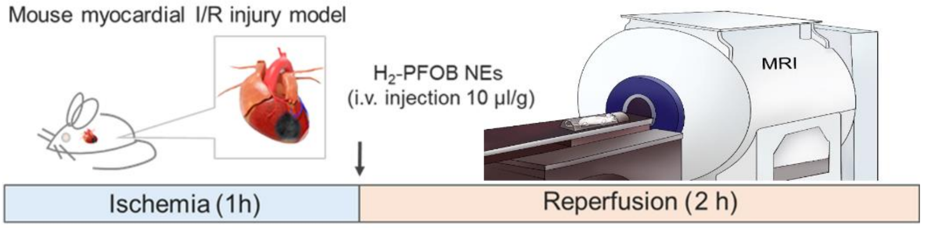

Supplementary Fig. 3. Cardiac ^{19}F-MRI imaging is performed 1 hour after myocardial ischemia in mice, which is also at the beginning of reperfusion. H_2-PFOB NEs are injected through the tail vein at the beginning of reperfusion (10μL/g 30% (v/v)).

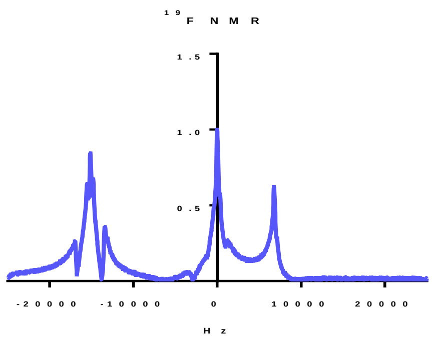

Supplementary Fig. 4. ^{19}F spectra of infiltrated H_2-PFOB NEs in the heart of I/R mice was obtained after injection of H_2-PFOB NEs.

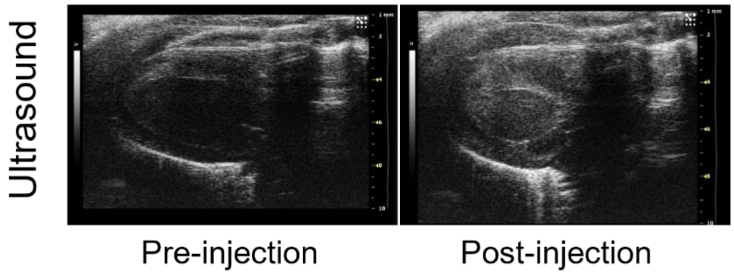

Supplementary Fig. 5. US signals of H_2-PFOB NEs distribution in myocardium.

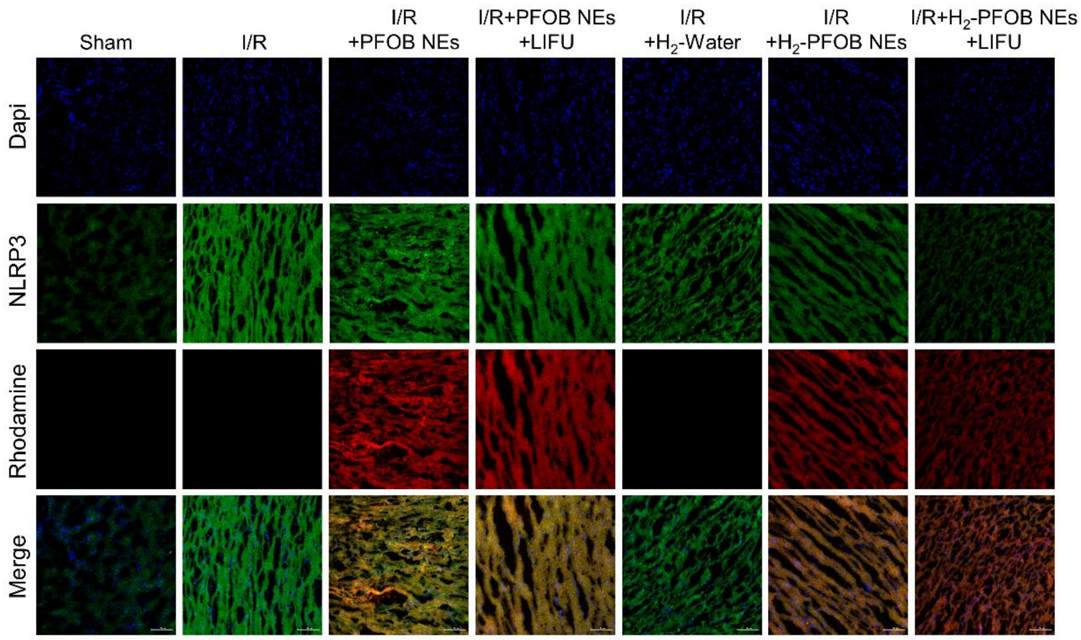

Supplementary Fig. 6. Representative NLRP3 immunofluorescence images in each group.

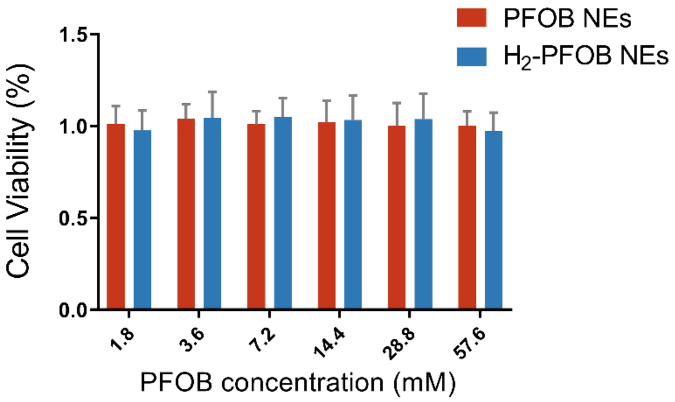

Supplementary Fig. 7. Cell viabilities of XX cells after co-incubation with H_2-PFOB NEs or PFOB NEs, n=6. Data are shown by mean ± SD

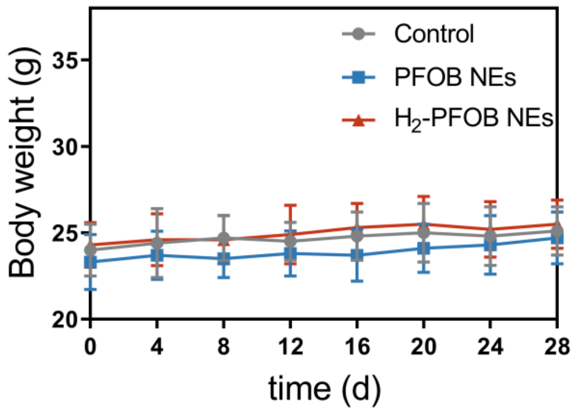

Supplementary Fig. 8. The body weight changes of mice after the intravenous injection of PFOB and H_2-PFOB, n=5. Data are shown by mean ± SD.

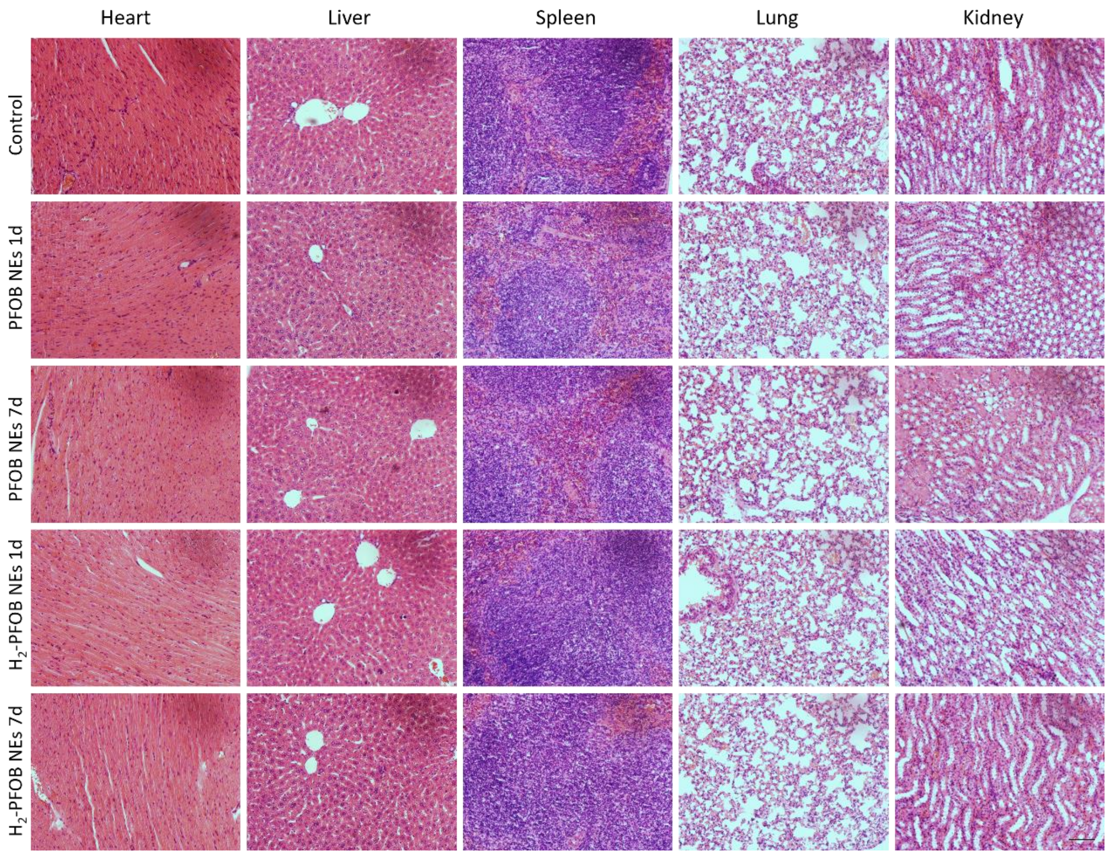

Supplementary Fig. 9. The results of HE staining of the effect of H_2-PFOB NEs on main organs (heart, liver, spleen, lung and kidney).

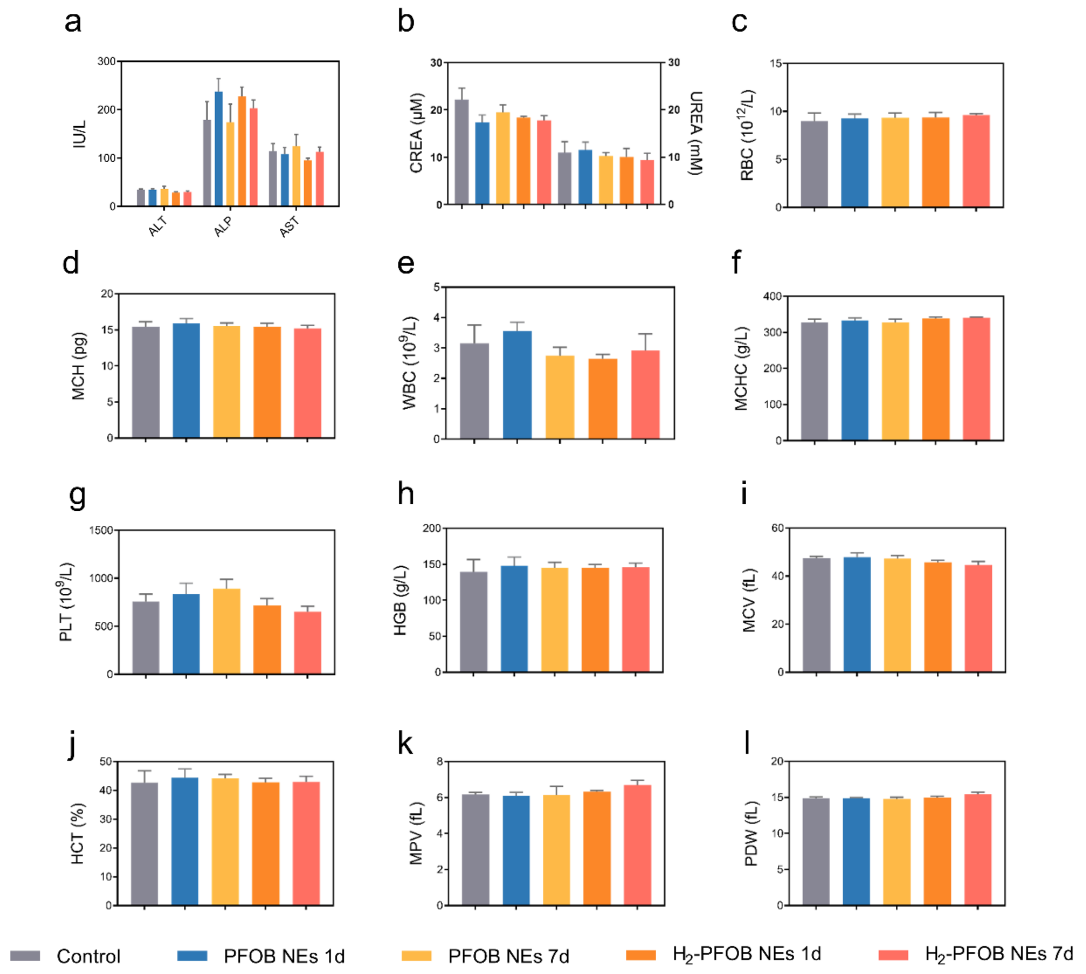

Supplementary Fig. 10. Effect of H_2-PFOB NEs on hematology markers. a CREA and UREA. b ALT, ALP and AST. c RBC. d MCH. e WBC. f MCHC. g PLT. h HBG. i MCV. j HCT. k MPV. l PDW. n=5, Data are shown by mean ± SD.
